# Outcome quality standards in advanced ovarian cancer surgery

**DOI:** 10.1186/s12957-020-02064-7

**Published:** 2020-11-25

**Authors:** Antoni Llueca, Anna Serra, Maria Teresa Climent, Blanca Segarra, Yasmine Maazouzi, Marta Soriano, Javier Escrig, on behalf MUAPOS Working Group, L. Gomez-Quiles, J. Escrig, A. Serra, K. Maiocchi, A. Villarin, M. Rodrigo-Aliaga, S. Martinez, C. Herrero, N. Ruiz, E. Izquierdo, V. Bosso, Y. Maazouzi, B. Segarra, M. C. Beltran, A. Llueca

**Affiliations:** 1 Department of Obstetrics and Gynecology, University General Hospital of Castellón, Av Benicasim s/n, 12004 Castellón, Spain; 2 Multidisciplinary Unit of Abdominal Pelvic Oncology Surgery (MUAPOS), University General Hospital of Castellón, Av Benicasim s/n, 12004 Castellón, Spain; 3 Department of Medicine, University Jaume I (UJI), Castellón, Spain; 4 Department of Anesthesiology, University General Hospital of Castellón, Av Benicasim s/n, 12004 Castellón, Spain; 5 Department of General Surgery, University General Hospital of Castellón, Av Benicasim s/n, 12004 Castellón, Spain

**Keywords:** Advanced ovarian cancer, Cytoreductive surgery, Outcome, Morbidity, Tumor, Quality indicator, Medical care

## Abstract

**Introduction:**

Advanced ovarian cancer surgery (AOCS) frequently results in serious postoperative complications. Because managing AOCS is difficult, some standards need to be established that allow surgeons to assess the quality of treatment provided and consider what aspects should improve. This study aimed to identify quality indicators (QIs) of clinical relevance and to establish their acceptable quality limits (i.e., standard) in AOCS.

**Materials and methods:**

We performed a systematic search on clinical practice guidelines, consensus conferences, and reviews on the outcome and quality of AOCS to identify which QIs have clinical relevance in AOCS. We then searched the literature (from January 2006 to December 2018) for each QI in combination with the keywords of advanced ovarian cancer, surgery, outcome, and oncology. Standards for each QI were determined by statistical process control techniques. The acceptable quality limits for each QI were defined as being within the limits of the 99.8% interval, which indicated a favorable outcome.

**Results:**

A total of 38 studies were included. The QIs selected for AOCS were complete removal of the tumor upon visual inspection (complete cytoreductive surgery), a residual tumor of < 1 cm (optimal cytoreductive surgery), a residual tumor of > 1 cm (suboptimal cytoreductive surgery), major morbidity, and 5-year survival. The rates of complete cytoreductive surgery, optimal cytoreductive surgery, suboptimal cytoreductive surgery, morbidity, and 5-year survival had quality limits of < 27%, < 23%, > 39%, > 33%, and < 27%, respectively.

**Conclusion:**

Our results provide a general view of clinical indicators for AOCS. Acceptable quality limits that can be considered as standards were established.

## Highlights

- Quality indicators are described in advanced ovarian cancer surgery(AOCS)
- Acceptable quality limits are defined as standards in AOCS

## Introduction

Advanced ovarian cancer surgery (AOCS) aims to achieve maximal cytoreduction to increase survival, and even provide a definitive cure in some cases. Generally, this comprises an aggressive surgery that frequently results in serious postoperative complications, including patient’s mortality or the impossibility of administration of subsequent oncological treatments. These complications can directly affect survival [1, 2].

A standard defines the range in which the level of quality reached in a certain process is acceptable, leading to establishment of the minimum allowable for an indicator. This implies comparing results in management of a specific disease through measurable, valid, and relevant indicators [3]. Because of the difficulty in managing advanced ovarian cancer (AOC), some standards need to be established that allow surgeons to assess the quality of the treatment provided and consider what aspects should improve, as well as compare their results with those of other groups. Investigation of quality surgical care is a multidimensional and complex challenge. The concept of quality may reflect different aspects of medical care, depending on the perspective under consideration. Structure (e.g., sufficient personnel, resuscitation cart availability, and working atmosphere), process (e.g., percentage of patients fulfilling the antibiotic prophylaxis protocol and patients with informed consent), and direct clinical outcomes (e.g., morbidity, mortality, and wound dehiscence) can be measured as a reflection of the quality of medical care [4].

Because of the complexity of AOCS, clinicians and surgeons require additional quality indicators (QIs) and their variability limits to determine what may be considered optimal or suboptimal treatment. This study aimed to identify QIs of clinical relevance and to establish their acceptable quality limits (AQLs) in AOCS according to a formal methodology.

## Materials and methods

### Search strategy

The research process was carried out in two parts. First, we needed to identify which QIs have clinical relevance in AOCS. Therefore, we performed a systematic search on clinical practice guidelines [5–7], consensus conferences [8], and reviews on the outcomes and quality of AOCS [9–15] that were published in MEDLINE/PubMed, Embase, and the Cochrane Library.

Second, once a series of QIs were selected from this literature, a new search was performed for each QI in combination with the Medical Subject Heading keywords of advanced ovarian cancer, ovary, surgery, and oncology. Only papers published from January 2006 to December 2018 were included in this part of the investigation. Additional relevant studies were also selected from the references that were obtained through the search. For inclusion in the analysis, each evaluated QI had to be clearly mentioned in the text or be easily calculated from the data presented in the study. When studies included series with oncological and non-oncological cases, careful selection of the results of the oncological cases was performed. When such selection was not possible, the paper was not included. When several studies were reported by the same institution, the study with the highest number of cases was selected. When studies included results from primary cytoreductive surgery and interval debulking surgery, careful selection of the primary cytoreductive surgery results was made. Studies were not included when such selection could not be performed. Despite being a retrospective review study of data from other authors, informed consent for the analysis was obtained from the Ethics and Research Committee (No. 02223/2019)

### Selection of QIs and studies

Indicators were selected according to clinical relevance (factors that clearly affected the prognosis or the postoperative course) and frequency of appearance in the different studies to ensure a sufficient amount of data for evaluation. Each QI was investigated individually. Therefore, there were different numbers of studies and cases for each QI, depending on the information available for each item in particular. Publications were only included in our study if they presented a minimum number of 50 cases for each item. Series that reported national or collected databases with thousands of patients were excluded because these studies did not clearly reflect the results of any particular team or institution.

### Data collection and determination of standards

Standards for each QI were determined by statistical process control (SPC) techniques. P-charts (categorical data) and X-charts (continuous data) were plotted to graphically represent the weighted average according to sample size as a quality standard. The studies were sorted in the charts according to the number of cases included in each series from a smaller to a larger volume of patients. According to Spiegelhalter et al. [16, 17] two limits represented by 99.8% confidence intervals (±3 standard deviations [SDs]) and 95% confidence intervals (±2 SDs) are calculated from the weighted average to establish variability limits. Any result outside of these limits significantly deviates from the weighted average (*p* < 0.002 and *p* < 0.05, respectively), and is considered out of control according to the SPC terminology. In our study, the AQLs for each QI were defined as being within the limits of the 99.8% interval, which indicated a favorable outcome. When a result was within the AQLs, it was considered to be within the standards.

## Results

### QIs

The QIs selected for AOCS were complete removal of the tumor upon visual inspection (complete cytoreductive surgery [CCS]), a residual tumor of < 1 cm (optimal cytoreductive surgery [OCS]), a residual tumor of > 1 cm (suboptimal cytoreductive surgery [SCS]) [2], major morbidity (grades III and IV of Clavien–Dindo classification) [18], and 5-year survival. A total of 38 studies were identified (Table 1) [19–54].

**Table 1:** Primary cytoreductive surgery results in advanced ovarian cancer

Author	N	CCS %	CCS Nº	OCS %	OCS Nº	SCS %	SCS Nº	Major complications %	5 Year survival %
Du Bois et al. 2009 [19]	3126	33.5	1046	31.2	975	35.3	1105		39
Bookman et al. 2009 [20]	4312	24.2	1044	45.1	1949	30.5	1319		
Chang et al. 2012 [21]	203	31	63	38	77	31	63	38	50
Chi et al. 2012 [22]	285	24	69	47	134	29	82	10	
Colombo et al. 2008 [23]	142	25.6	39	35.2	50	37.3	53	14	30
Fago-Olsen et al. 2014 [24]	990	39	381	27	266	35	341	19	30
Fagotti et al. 2013 [25]	148	62	92	27.7	41	10	15	8	30
Luyckx et al. 2012 [26]	527	71	374	18	94	11	57		50
Peiretti et al. 2010 [27]	288	44	115	32	83	24	61	16	49
Rauh-Hain et al. 2012 [28]	176	7.5	13	58	102	34	61	30	20
Rauh-Hain et al. 2013 [29]	330	26	85	56	187	17.6	58		
Rosen et al. 2014 [30]	143	41.5	76	22.4	41	35.5	65		50
Sehouli et al. 2010 [31]	332	59.8	196	27.4	90	12.5	41	36.5	39
Tropé et al. 2012 [32]	127	14	18	36	44	50	62	31	12
Vergote et al. 2010 [1, 33]	336	19	64	22	70	60	202	22	20
Winter et al. 2008 [34]	330	8	29	21.7	71	251	76		20
Amstrong IV 2006	210	36	75			64	135		50
Amstrong IP 2006	205	38	78			62	127		
Braicu et al. 2011 [36]	415							33.5	
Eisenkop et al. 2003 [37]	408	86	351	10	41	4	16		
Chi et al. 2009 [38]	210	27	57	52	110	20	43	19	
Eisenhauer1	57	23	13	77	44	0	0	12	
Eisenhauer2	122	30	37	70	85	0	0	7	
Chi et al. 2006 [40]	441	15	67	36	169	49	233		65
Chi et al. 2010 [41]	141	30	42	60	85	10	14	22	48
Kobal et al. 2018 [42]	108	54	58	17.6	19	29	31	22	36
Kang et al. 2011 [43]	140	58	81	33.6	47	8.6	12		
Kommos 2010 [44]	267	47	126	32.6	87	20	57	33	
Magtibay et al. 2006 [45]	66	9	6	32	21	18	12	28	40
Rafii et al. 2012 [46]	180							11	
Rodriguez et al. 2013 [47]	4312	20	860	40	1746	39	1647		40
Salani et al. 2007 [48]	102	39	40					47	35
Sperling et al. 2013 [49]	3129	46	1296			54	1502		40
Van Meurs et al. 2013 [50]	336								20
Wimberger et al. 2010 [51]	573	12		29		58			
Winter et al. 2007 [52]	1895	23	437	41	791	35	667		40
Zivanovic et al. 2010 [53]	526	18	93	42	221	40	212		
Llueca and Escrig 2017 [54]	90	82	74	6	5	14	11	31	39
**Total patients**	**25728**								

### Cytoreductive rate

**Fig. 1:**
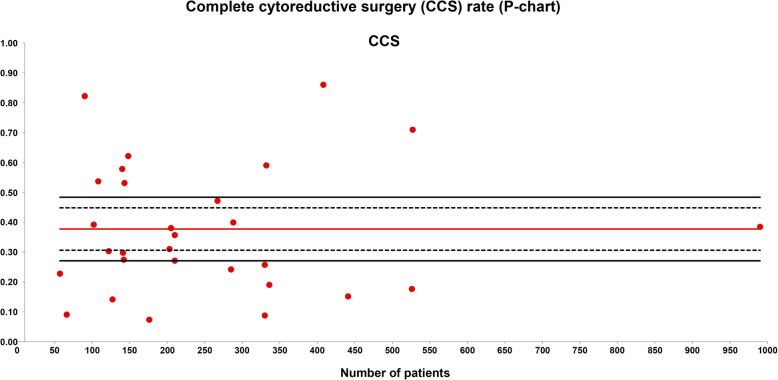
Complete cytoreductive surgery (CCS) rate (P-chart). Each dot represents an included study. The gray area (standard zone) is within the 95% confidence interval, the blue area (alert zone) is between the 95% and 99.8% confidence intervals, and the white area (alarm zone) is outside the 99.8% limit

The CCS rate was evaluated in 28 series with a total of 7450 patients included. The weighted average CCS was 38% and the quality limit was < 27% (Fig. 1).

**Fig. 2:**
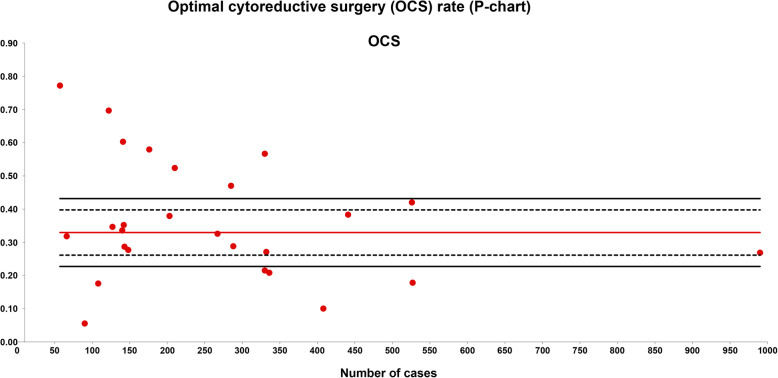
Optimal cytoreductive surgery (OCS) rate (P-chart). Each dot represents an included study. The gray area (standard zone) is within the 95% confidence interval, the blue area (alert zone) is between the 95% and 99.8% confidence intervals, and the white area (alarm zone) is outside of the 99.8% limit

The OCS rate was evaluated in 26 series with a total of 6933 patients included. The weighted average OCS was 33% and the quality limit was < 23% (Fig. 2).

**Fig. 3:**
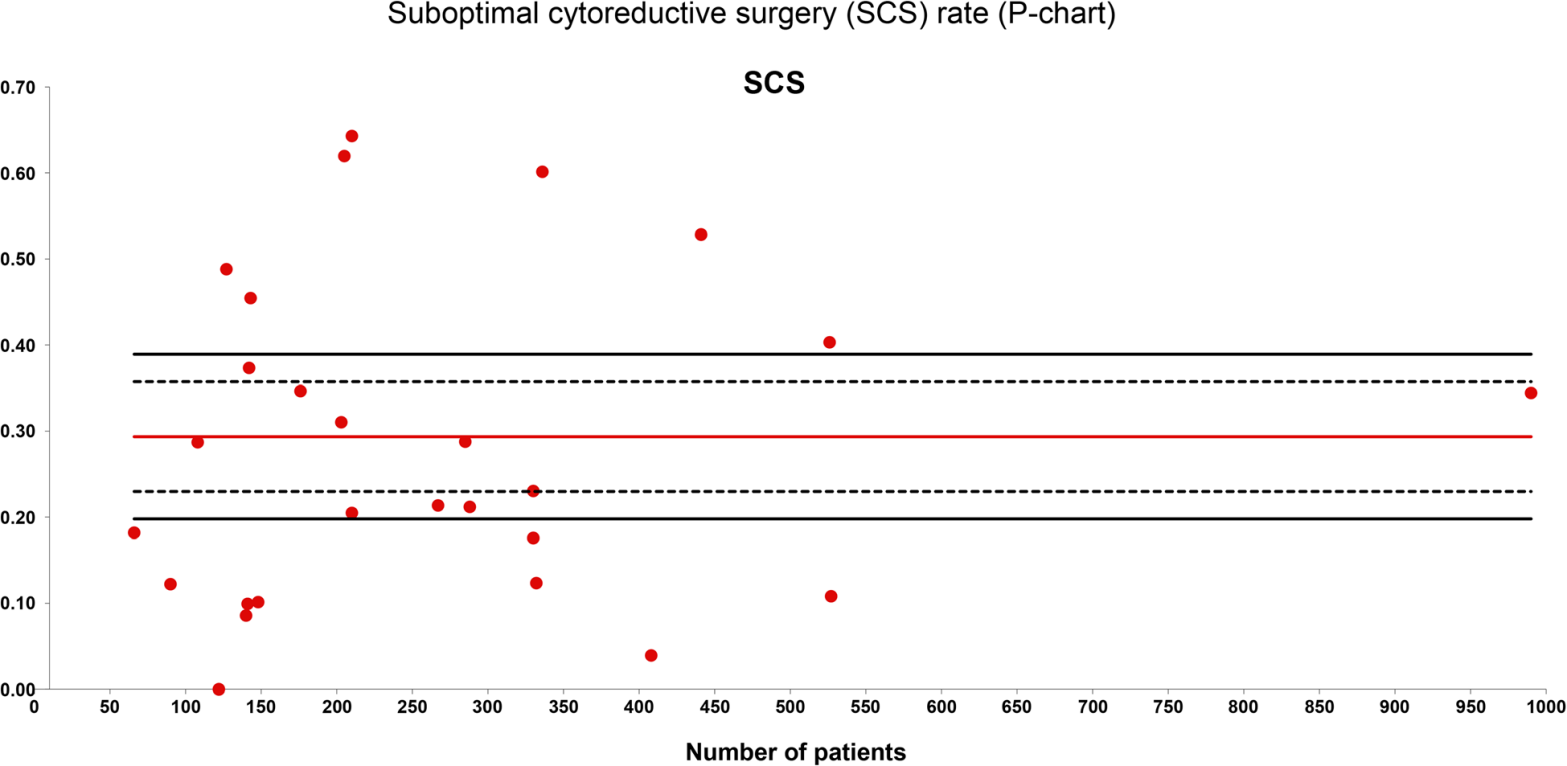
Suboptimal cytoreductive surgery (SCS) rate (P-chart). Each dot represents an included study. The gray area (standard zone) is within the 95% confidence interval, the blue area (alert zone) is between the 95% and 99.8% confidence intervals, and the white area (alarm zone) is outside of the 99.8% limit

The SCS rate was evaluated in 27 series with a total of 7291 patients included. The weighted average SCS was 29% and the quality limit was > 39% (Fig. 3).

### Morbidity

**Fig. 4:**
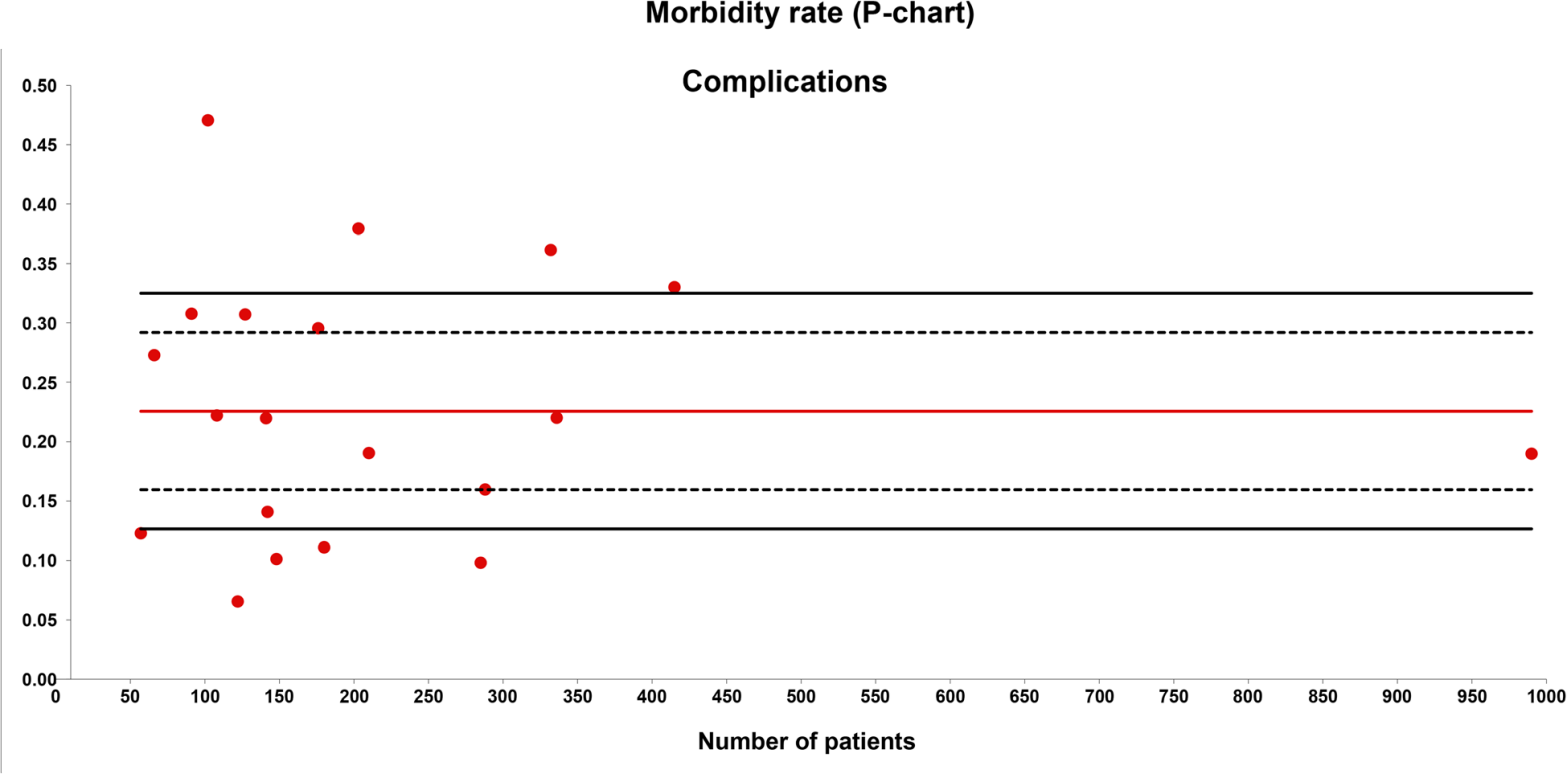
Morbidity rate (P-chart). Each dot represents an included study. The gray area (standard zone) is within the 95% confidence interval, the blue area (alert zone) is between the 95% and 99.8% confidence intervals, and the white area (alarm zone) is outside of the 99.8% limit

Major complications were evaluated in 23 series with a total of 4519 patients evaluated. The weighted average morbidity was 23% and the quality limit was > 33% (Fig. 4).

### Survival

**Fig. 5:**
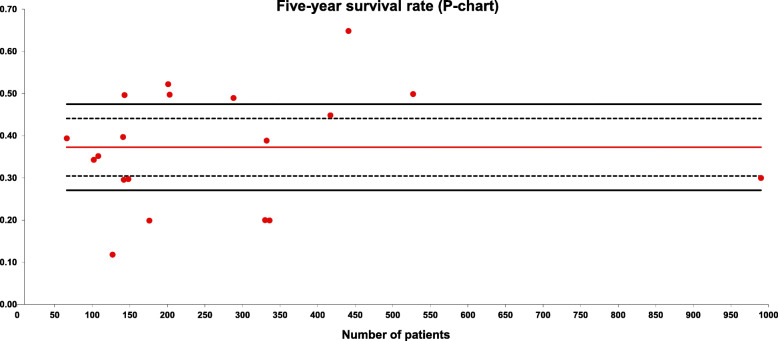
Five-year survival rate (P-chart). Each dot represents an included study. The gray area (standard zone) is within the 95% confidence interval, the blue area (alert zone) is between the 95% and 99.8% confidence intervals, and the white area (alarm zone) is outside of the 99.8% limit

The 5-year survival rate was evaluated in 19 series with a total of 5554 patients included. The weighted average overall survival (OS) was 37% and the quality limit was < 27% (Fig. 5).

## Discussion

A QI is a parameter that is used to measure the quality of medical assistance and provides information on aspects that can be improved. To evaluate the quality of a service or process, comparisons need to be established with some sort of guide or reference. This reference is what we define as a standard. Ideally, quality should be measured for populations with a common health care system and similar social customs (i.e., those in which different surgical teams have similar access to resources). Moreover, any risk-adjusted evaluation of the parameters involved in measuring quality should be taken into account for comparisons. In the absence of these ideal conditions, standards need to be identified in the data available in the literature, even if this means comparing systems as different as those in Japan, Europe, or the USA. This will at least offer a global perspective of what standards should be considered. Several studies have demonstrated the importance of hospital volume and mortality associated with treatments, and they have made important contributions to our understanding of the quality of surgical care. However, these parameters are insufficient [55, 56].

In 2016, the European Society of Gynaecological Oncology (ESGO) published an assessment of advanced ovarian cancer care based on formally developed QIs [9]. In this report, 10 QIs, including structural factors, clinical care process, treatment efficacy, and outcomes, were described. However quality limits where established only in two QIs, including the rate of complete surgical resection and the rate of primary debulking surgery. The quality limits were > 50% for both QIs. There are important methodological differences between the study by the ESGO and our study. The most relevant difference between studies is that the study by the ESGO identified QIs for a variety of factors based on estimations of a panel of ovarian cancer experts who were more focused on the accreditation of reference centers and health planning of ovarian cancer care. Our study identified outcome QIs and established AQLs for all items according to worldwide data. We used a different statistical methodology aimed to control variability that is inherent in the data of different institutions. Because these two studies covered different aspects of quality health care and methodology, they can be considered complementary.

An investigation such as our study has several disadvantages when selecting QIs and series. With regard to QIs, there may be other indicators (e.g., operative time, blood transfusion, length of hospital stay, and costs). However, our selection was based on the most frequently published data available to obtain a relevant volume of series and cases for analysis. We used literature that was published in English between 2006 and 2018 for selection of the series. From 2005, there has been worldwide development and progress in this highly complex AOCS. We believe that our results offer a general view of what the clinical indicators should be, as well as establishing AQLs that can be considered as standards.

In our study, standards and AQLs were obtained using weighted averages and confidence intervals of 99.8% and 95%. Using this method, the probability of a particular observation being outside such limits is low if the evaluated procedure is under control (i.e., *p* < 0.002 and *p* < 0.05). In SPC terminology, such limits establish alarm (99.8%) or alert (95%) areas. When a result is either above or below the alarm zone, it may be considered either unfavorable or outstanding, depending on the nature of the evaluated variable. For CCS, a result below the alarm zone limit should be considered a bad outcome. However, for morbidity, when a result is below the alarm zone limit, it is considered to be an excellent result.

The CCS rates reported in the literature are highly variable, not only between regions, but within the same region. It is a common phenomenon that depends on the specialization of the working groups and their multidisciplinary organization. In our study, OCS and CCS quality limits were 71% in AOCS when considered together. This result is similar to that published recently by Park et al. where the percentage of OCS and CCS was 78% in the responses of a survey [57].

In our study, almost all of the graphs showed great variability of the results of the series that were investigated. This is called statistical overdispersion of the data [58]. The statistical phenomenon of overdispersion can be explained by two reasons. First, this phenomenon can be caused by the different definitions used in the studies, which may result in a case selection bias. Second, and much more likely, is that we did not compare similar practices.

In clinical practice, the FIGO (International Federation of Gynecology and Obstetrics) system is widely used to characterize and predict survival in ovarian cancer [59]. Intraperitoneal spread is the most typical presentation of stages III and IV ovarian cancer. Unfortunately, the FIGO system fails in characterization of tumor burden and in describing anatomical regions that are affected. Patients with stage IIIc ovarian cancer may have localized and easily resectable carcinomatosis, but they may also have extensive unresectable disease [60]. To objectively determine and quantify the tumor burden in these advanced ovarian cancer stages, some authors have described assessment tools in the field of surgical oncology. One of the most frequently used tool is the Peritoneal Cancer Index (PCI), which describes peritoneal carcinomatosis of all types [61]. Specifically for ovarian cancer, Fagotty, Allety, and Zivanovic [54] described some other assessment tools. The PCI provides useful information about the precise distribution and tumor volume, representing in detail the extent of peritoneal spread. We believe that the PCI should be incorporated as a complement to the FIGO stages for comparison of different series of different authors in a more objective manner. This process could decrease the rate of overdispersion of QIs.

In the same manner, drawing conclusions about morbidity is difficult if we are not speaking the same language. The overdispersion phenomenon is also observed between the lower and the upper quality limits of the morbidity rate in a P-chart. Recently, we found that major complications (grades III–IV of Clavien–Dindo) were much higher with a PCI >20 than those found with a PCI < 10 [2]. This finding could explain the enormous variability of data observed for the morbidity rate (Fig. 4). However, the aggressiveness of the tumor and the tumor biology itself could have some relevant role in this matter.

OS and progression-free survival (PFS) are important endpoints for evaluating new anti-cancer therapies. Although OS is considered to be the most clinically relevant endpoint in ovarian cancer treatment, it is affected by use of multiple post-progression therapies. Additionally, the time required to evaluate OS is often considerable. PFS, which measures the time to disease progression or death after treatment, is a useful endpoint because it can show a clinical benefit and is more rapidly assessable than OS [62]. Although the use of PFS is increasing considerably, most authors still refer to OS as a reference value. There are many factors that influence OS. In our study, the average 5-year OS rate was 37%, and there was a large amount of variability (Fig. 5). As previously reported, the OS rate can rise to > 50% with a PCI < 10 and the inverse situation occurs with a PCI > 20 [63, 64].

This study has several limitations. First, this was a narrative retrospective review that combined data from clinical trials and retrospective institutional cohorts. Therefore, conclusions must be drawn with caution. Second, extracting data from the published series was difficult because of the enormous variability of the variables and the concepts included in the series. Therefore, there may have been some interpretation bias. Variability is inherent to any process, although beyond a certain variability, it becomes excessive. The key point to dealing with variability is delimiting and distinguishing normal and undesirable variability. The method used in this study allowed us to define the interval that discriminates variability from dispersion of data.

In conclusion, for advanced ovarian cancer surgery, five QIs were identified, the rates of CCS, OCS, SCS, complications and overall 5-year survival with AQLs of < 27%, < 23%, > 39%, > 33%, and < 27%, respectively. For most physicians and surgeons, quality indicates good clinical outcomes and the effectiveness of treatment or surgery. In the clinical setting, quality should be measured according to objective clinical indicators, which was the goal of our study. Specific values that define quality standards in AOCS are essential.

## Data Availability

Anonymized data can be provided upon reasonable request to corresponding author.
