# Correction to: Outcome quality standards in advanced ovarian cancer surgery

**DOI:** 10.1186/s12957-020-02102-4

**Published:** 2020-12-07

**Authors:** Antoni Llueca, Anna Serra, Maria Teresa Climent, Blanca Segarra, Yasmine Maazouzi, Marta Soriano, Javier Escrig, on behalf MUAPOS Working Group

**Affiliations:** 1 Department of Obstetrics and Gynecology, University General Hospital of Castellón, Av Benicasim s/n, 12004 Castellón, Spain; 2 Multidisciplinary Unit of Abdominal Pelvic Oncology Surgery (MUAPOS), University General Hospital of Castellón, Av Benicasim s/n, 12004 Castellón, Spain; 3 Department of Medicine, University Jaume I (UJI), Castellón, Spain; 4 Department of Anesthesiology, University General Hospital of Castellón, Av Benicasim s/n, 12004 Castellón, Spain; 5 Department of General Surgery, University General Hospital of Castellón, Av Benicasim s/n, 12004 Castellón, Spain

**Correction to: World J Surg Oncol (2020) 18:309**

**https://doi.org/10.1186/s12957-020-02064-7**

Following publication of the original article [1], the authors identified an error in the Figures. The figures have been incorrectly cropped at the right part during production processing.

The correct figures have been updated in the original article.
